# Artificial antigen-presenting cells expressing AFP_158-166_ peptide and interleukin-15 activate AFP-specific cytotoxic T lymphocytes

**DOI:** 10.18632/oncotarget.8198

**Published:** 2016-03-19

**Authors:** Longhao Sun, Hao Guo, Ruoyu Jiang, Li Lu, Tong Liu, Zhixiang Zhang, Xianghui He

**Affiliations:** ^1^ Department of General Surgery, Tianjin Medical University General Hospital, Tianjin, China

**Keywords:** artificial antigen-presenting cells, alpha-fetoprotein, interleukin-15, cytotoxic T lymphocytes, adoptive immunotherapy, Immunology and Microbiology Section, Immune response, Immunity

## Abstract

Professional antigen-presenting cells (APCs) are potent generators of tumor antigen-specific cytotoxic T lymphocytes (CTLs) for adoptive immunotherapy; however, generation of APCs is cumbersome, expensive, and subject to the tumor microenvironment. Artificial APCs (aAPCs) have been developed as a cost-effective alternative to APCs. We developed a cellular aAPC that efficiently generated alpha-fetoprotein (AFP)-specific CTLs. We genetically modified the human B cell lymphoma cell line BJAB with a lentiviral vector to establish an aAPC called BA15. The expression of AFP_158-166_-HLA-A*02:01 complex, CD80, CD86, and interleukin (IL)-15 in BA15 cells was assessed. The efficiency of BA15 at generating AFP-specific CTLs and the specific cytotoxicity of CTLs against AFP+ cells were also determined. BA15 cells expressed high levels of AFP_158-166_ peptide, HLA-A2, CD80, CD86, and IL-15. BA15 cells also exhibited higher efficiency in generating AFP-specific CTLs than did dendritic cells. These CTLs had greater cytotoxicity against AFP+ hepatocellular carcinoma cells than did CTLs obtained from dendritic cells *in vitro* and *in vivo*. Our novel aAPC system could provide a robust platform for the generation of functional AFP-specific CTLs for adoptive immunotherapy of hepatocellular carcinoma.

## INTRODUCTION

Adoptive cellular immunotherapy with antigen-specific cytotoxic T lymphocytes (CTLs), which utilizes the power and specificity of the autologous immune system, has emerged as a promising strategy for the treatment of established malignancies [1–5]. For effective therapeutic infusion, adequate quantities of *in vitro*-primed and -expanded high-affinity T cells restricted to tumor-associated antigens are necessary. Antigen-presenting cells (APCs), particularly dendritic cells (DCs), macrophages, and B lymphocytes, are the most potent stimulators of the generation and amplification of tumor antigen-specific CTLs in adequate quantities for clinical use [6–8]. However, the isolation and generation of sufficient autologous APCs is cumbersome and expensive, requiring a large amount of blood from patients or compatible healthy donors. Moreover, the efficiency of APCs in inducing and expanding antigen-specific T cells is significantly affected by tumor microenvironments [9].

Previous studies have demonstrated that the epitope peptide FMNKFIYEI (AFP_158-166_) is a HLA-A*0201-restricted human hepatocellular carcinoma (HCC)-specific antigen that can stimulate specific T cell responses [10]. The main obstacle limiting the wide application of alpha-fetoprotein (AFP)-specific CTLs in adoptive immunotherapy is the technical challenge of manufacturing them. Currently, the generation of CTLs requires several rounds of activation with antigenic peptide-pulsed DCs and a high dosage of interleukin (IL)-2, which are time-consuming and expensive procedures. Moreover, the efficiency of the specific activation is quite low.

Artificial antigen-presenting cells (aAPCs) have been developed as a cost-effective alternative to natural APCs [11, 12]. aAPCs are engineered using a wide variety of platforms and are readily prepared as off-the-shelf, standardized, and renewable reagents that deliver the appropriate signals to naive T cells. Cellular aAPCs are commonly based on xenogeneic or allogeneic cells and genetically modified using retrovirus or lentivirus transduction [13–16]. Also, acellular aAPCs can be synthesized by covalently coupling the MHC/peptide complex and CD28 ligand on various materials [17]. However, in most of the currently available aAPC systems, synthetic antigenic peptides are exogenously loaded on to aAPCs. In general, exogenous peptides are processed by APCs in the endosomes and presented to CD4+ T cells by MHC class II molecules through the class II pathway, while endogenously expressed peptides are processed in the cytoplasm and presented to CTLs by MHC class I molecules through the class I pathway. Although there are interconnections and cross-presentations between the class I and class II pathways, the efficiency of CTL activation and expansion is reduced when exogenous loading methods are used [18].

In the current study, we employed human B cell lymphoma cell line BJAB as the scaffold to develop a cellular aAPC that could efficiently generate AFP-specific CTLs for adoptive immunotherapy of HCC [19]. BJAB cells were chosen because they exhibit a high expression level of adhesion molecules, including CD80 and CD86, which are known as the co-stimulatory signals in T cell activation and are easy to handle for cell proliferation and genetic modification. BJAB cells were genetically engineered to endogenously express both AFP_158-166_ peptide and IL-15 *via* lentivirus transduction, which we expected would increase the specific activation rate of AFP_158-166_-specific CTLs. We then conducted a series of function tests on the resulting BA15 cells to evaluate the specific cytotoxicity of CTLs against HCC cells *in vitro* and *in vivo*.

## RESULTS

### Establishment of aAPC expressing peptide-MHC complex, co-stimulatory molecule ligands, and cytokine

**Figure 1:**
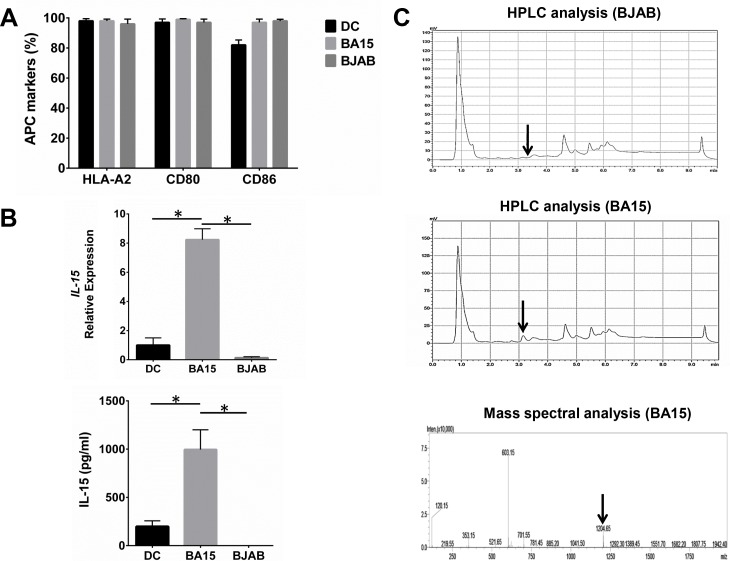
Expression of AFP_158-166_ peptide-HLA-A*02:01 complex, CD80, CD86, and IL-15 in DC, BA15, and BJAB cells **A.** FCM revealed that there were not significant differences in the expression of HLA-A2, CD80, and CD86 among DC, BA15 and BJAB cells. **B.** qRT-PCR and ELISA showed that the mRNA and secreted protein levels of IL-15 in BA15 cells were significantly higher than those in DC and BJAB cells. **C.** HPLC showed that the eluting peak corresponding to the synthetic AFP_158-166_ peptide was found in acid-stripped BA15 cells but not in BJAB cells. Mass spectrometry also revealed that the molecular weight of the peptide in this eluting peak was the same as that of the synthetic peptide. Error bars indicate standard deviations. * indicates *P* < 0.05.

HLA-A2, CD80, and CD86 were expressed at high levels in BA15 cells because these cells are based on the BJAB cell line. We found no significant differences in the expression of these proteins in BA15 cells compared with DC and BJAB cells (Figure 1A). Real-time quantitative PCR (qRT-PCR) and ELISA showed that the mRNA and secreted protein levels of IL-15 in BA15 cells were significantly higher than those in DC and BJAB cells (Figure 1B). The presence of the AFP_158-166_ peptide was biochemically confirmed directly from the HLA-A*02:01 groove on the surface of the BA15 cells. The high-performance liquid chromatography (HPLC) analysis revealed that an eluting peak corresponding to the synthetic peptide was found in acid stripping of BA15 cells, but not in BJAB cells. Mass spectrometry revealed that the molecular weight of the peptide in this eluting peak was the same as that of the synthetic peptide (Figure 1C).

### Stability of peptide-MHC complex, co-stimulatory molecule ligands, and cytokine expression in aAPCs after γ-ray irradiation

**Figure 2:**
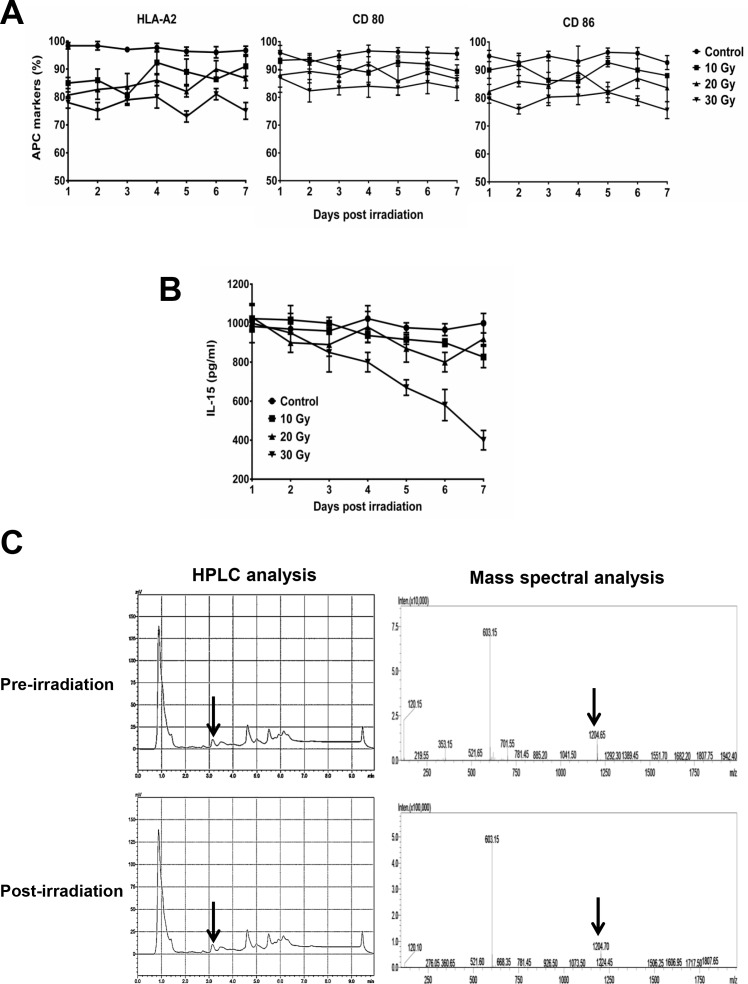
Stability of AFP_158-166_ peptide-HLA-A*02:01 complex, CD80, CD86, and IL-15 expression in BA15 cells after γ-ray irradiation **A.** FCM revealed that the expression of HLA-A2, CD80, and CD86 were not significantly affected by different dosages of irradiation. **B.** ELISA showed that the secretion of IL-15 in BA15 cells decreased after exposure to 30 Gy of irradiation but was stable at lower dosages. **C.** HPLC showed that the eluting peak corresponding to the synthetic AFP_158-166_ peptide was found in acid-stripped BA15 cells both pre- and post-irradiation. Mass spectrometry revealed that the molecular weight of the peptide in this eluting peak was the same as that of the synthetic peptide. Error bars indicate standard deviations.

In BA15 cells, the expression of HLA-A2, CD80, and CD86 were not significantly affected by different dosages of irradiation (Figure 2A). ELISA showed that the secretion of IL-15 in BA15 cells decreased after exposure to 30 Gy of radiation but was not significantly affected by irradiation at lower dosages (Figure 2B). HPLC showed that the eluting peak corresponding to the synthetic AFP_158-166_ peptide was found in acid-stripped BA15 cells both before and after treatment with 30 Gy of radiation. Mass spectrometry revealed that the molecular weight of the peptide in this eluting peak was the same as that of the synthetic peptide (Figure 2C).

### Inhibiting proliferation and inducing apoptosis of aAPCs by γ-ray irradiation

**Figure 3:**
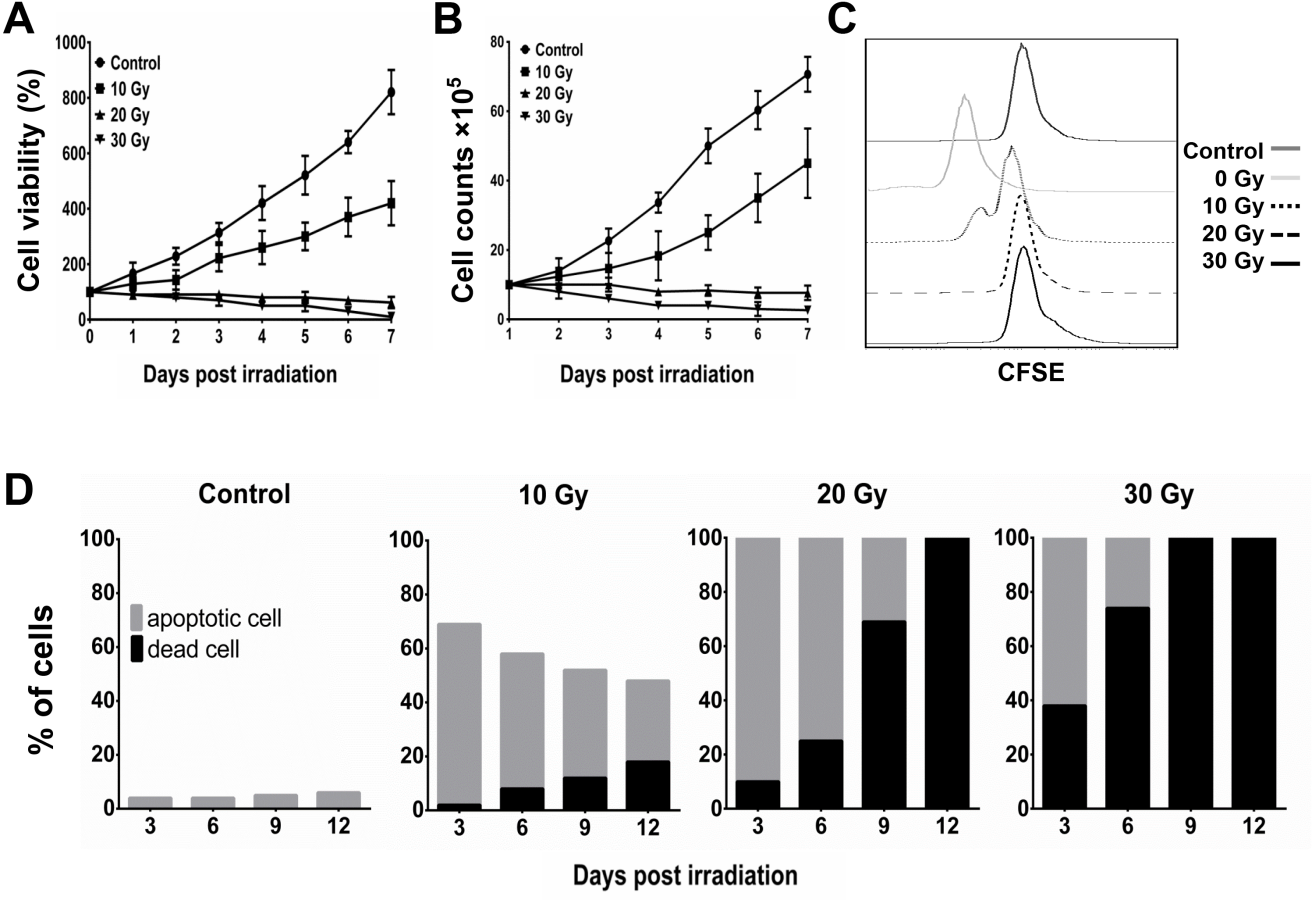
Inhibition of proliferation and induction of apoptosis of BA15 by γ-ray irradiation After different dosages of irradiation, the cell viability and proliferation of BA15 cells were analyzed by MTT, cell counting, and CFSE assays. Apoptosis assays were performed every 3 days after irradiation. **A.** MTT assay indicated that the cell viability of BA15 cells decreased after exposure to 20 Gy and 30 Gy of irradiation. **B.** Cell counting indicated that the number of BA15 cells decreased after exposure to 20 Gy and 30 Gy of irradiation. **C.** CFSE labeling revealed that the proliferation of BA15 cells was completely inhibited after exposure to irradiation of 20 Gy and 30 Gy. **D.** The apoptosis assay revealed that all the cells in the 20-Gy and 30-Gy group were in apoptosis or dead 3 days after irradiation and that all the cells had died by day 12. There were fewer dead cells in the 20-Gy group than in the 30-Gy group at every time point. Error bars indicate standard deviations.

In our dosage-course experiment using γ-ray irradiation, the MTT assay indicated that the viability of BA15 cells decreased after exposure to 20 Gy and 30 Gy of radiation (Figure 3A). The cell counting and carboxyfluorescein succinimidyl ester (CFSE) analyses indicated that BA15 cell proliferation was completely inhibited at doses of 20 Gy and 30 Gy (Figure 3B and 3C). Apoptosis assays performed every 3 days after irradiation for 12 days revealed that all the cells in the 20-Gy and 30-Gy groups were either in apoptosis or dead after irradiation; all the cells had died within 12 days. There were fewer dead cells in the 20-Gy group than in the 30-Gy group at each time point (Figure 3D). Thus, 20 Gy was determined to be the optimal dosage at which the proliferation of BA15 cells was completely inhibited while leaving most of the cells still viable within the frame of 1 round of activation (7 days). Expression of HLA-A2, CD86, CD80, IL-15, and AFP_158-166_ peptide was not significantly affected by radiation at that point. After the activation process, all BA15 cells would have to die to guarantee the clinical safety of adoptive infusion.

### Efficient activation and expansion of AFP_158-166_-specific CTLs by aAPCs

**Figure 4:**
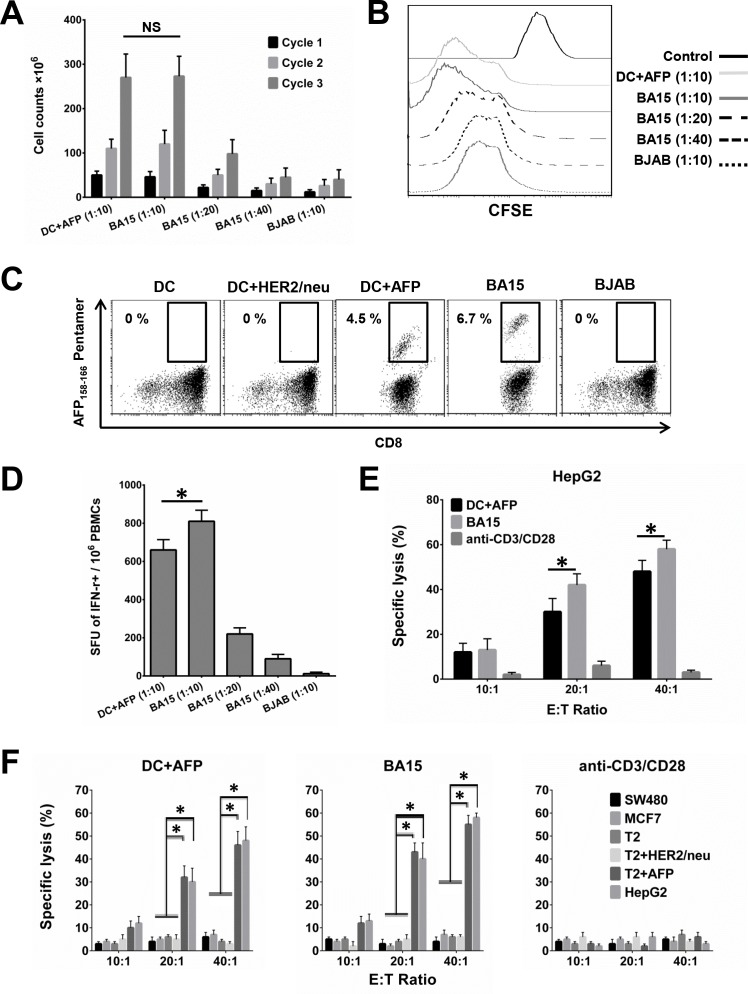
Activation and expansion of functional AFP_158-166_-specific CTLs with BA15 cells CTLs isolated from HLA-A*02:01+ healthy donors were stimulated weekly by co-culturing with different APCs for 3 cycles. **A.** Cell counting showing CTL activation by different APCs and APC/lymphocyte ratios. BA15 cells activated CTLs at maximum efficiency at a ratio of 1:10. After 3 cycles of stimulation at this ratio, BA15 cells and DCs showed the same CTL activation efficiency. **B.** CFSE assay showing activation of CTLs by different APCs at different APC/lymphocyte ratios. BA15 cells had maximum CTL activation efficiency at a ratio of 1:10. **C.** AFP_158-166_ MHC Pentamer staining showed that CTLs generated by BA15 cells established a higher proportion of AFP_158-166_-specific CTL population than CTLs generated by peptide-pulsed DCs (6.7 ±0.4% *vs*. 4.5 ±0.3%, respectively, *P* < 0.05). **D.** Secretion of IFN-γ by antigen-specific CTLs activated by different APCs at different APC/lymphocyte ratios. A ratio of 1:10 displayed the highest levels of antigen-specific secretion of IFN-γ. The mean number of SFU per million was 810.1 (±58.3) in BA15-activated CTLs *versus* 660.4 (±54.2) in CTLs obtained with AFP-pulsed DCs. **E.** Comparison of the specific lysis rate of CTLs activated by different APCs toward HepG2. The CTLs activated by BA15 cells had significantly higher lysis rates toward HepG2 cells than did the CTLs obtained from AFP-pulsed DCs. **F.** Comparison of the specific lysis rate of AFP-positive (HepG2 and T2+AFP) and AFP-negative (SW480, MCF7, T2, and T2+HER2/neu) cells. AFP-positive cells were specifically lysed by antigen-specific CTLs stimulated with BA15 and peptide-pulsed DCs, but AFP-negative cells were not. E: T Ratio indicates effector to target ratio. Error bars indicate standard deviations. * indicates *P* < 0.05. NS: not significant.

CTLs isolated from HLA-A*02:01+ healthy donors were stimulated by co-culturing with different APCs for 3 weekly cycles. Cell counting and CFSE assays showed that BA15 cells efficiently activated CTLs at different APC/lymphocyte ratios (1:10 and 1:20), with maximum efficiency at 1:10 (Figure 4A and 4B). After 3 weekly rounds of stimulation at this ratio, BA15 cells showed the same activation efficiency as DCs, but AFP_158-166_ MHC Pentamer staining showed that the percentage of AFP-specific CTLs was significantly higher in the BA15 cell population than in the DCs (6.7 ±0.4% *vs*. 4.5 ±0.3%, *P* < 0.05) (Figure 4C). The results indicated that the density of the AFP peptide expressed and presented by the HLA-A*02:01 molecule on BA15 cells was higher than that of the DC-pulsed exogenous AFP peptide (40 mg/mL). Furthermore, BJAB cells exhibited no significant CTL activation function.

### Functional tests of AFP_158-166_-specific CTLs expanded by aAPCs

To confirm the functionality of the antigen-specific CTLs, we assessed the IFN-γ secretion of CTLs that had been expanded by different APCs. The numbers of IFN-γ-secreting T cells differed across the APC-stimulated CTL groups and corresponded with the results determined by the AFP_158-166_ MHC Pentamer labeling. The antigen-specific CTLs activated by BA15 at the APC/lymphocyte ratio of 1:10 exhibited the highest rates of antigen-specific secretion of IFN-γ (Figure 4D). The mean number of spot-forming units (SFU) per million was 810.1 (±58.3) in BA15-activated CTLs *versus* 660.4 (±54.2) in CTLs obtained with AFP-pulsed DCs.

When we examined the cytotoxicity of the AFP-specific CTLs we generated, we found that AFP-positive cells (HepG2 and T2+AFP) but not AFP-negative cells (SW480, MCF7, T2, and T2+HER2/neu) were specifically lysed by antigen-specific CTLs stimulated with BA15 and peptide-pulsed DCs. This result suggests that the generated CTLs had sufficiently high antigen-specific T-cell receptor avidity to recognize AFP+ HCC cells. Furthermore, the CTLs activated by BA15 also had a higher specific lysis rate toward HepG2 cells than did the CTLs obtained from AFP-pulsed DCs. These results show that the CTLs generated by BA15 were able to recognize AFP-positive HCC cells and possessed potent cytotoxic functions (Figure 4E and 4F).

### AFP_158-166_-specific CTLs expanded by aAPCs suppress tumor growth in tumor-bearing NOD /SCID mice

**Figure 5:**
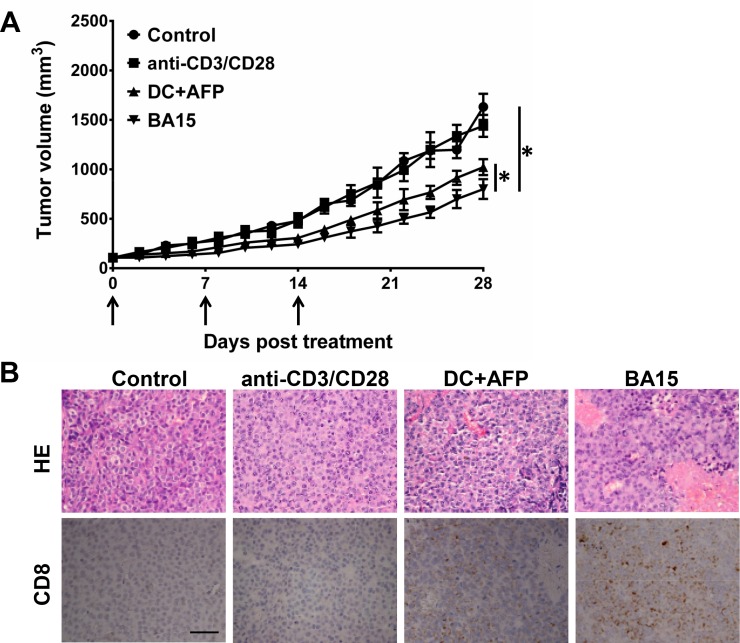
Suppression of tumor growth *in vivo* by AFP_158-166_-specific CTLs generated by BA15 cells HepG2 cells were used to establish xenograft tumors in NOD /SCID mice. Nonspecific activated CTLs, DCs, or BA15-induced AFP-specific CTLs (1 × 10^8^ in 0.2 mL PBS) were transferred into mice of different groups *via* intravenous injection at days 0, 7, and 14. Black arrows indicate the treatment times. PBS-treated mice were used as a control group. **A.** Treatment with AFP-specific CTLs expanded by DCs and BA15 displayed significantly higher tumor-suppressive functions than treatment with nonspecific activated CTLs or PBS. CTLs expanded by BA15 were the most efficient of these groups. **B.** HE staining showed that there were significantly more tumor cells exhibiting morphological changes indicative of apoptosis and necrosis in the BA15 group than others. The infiltration of CD8+ CTLs in the BA15 group was also significantly higher than that in other groups (*P* < 0.01). Scale bar = 100 μm; magnification, 400 ×. Error bars indicate standard deviations. * indicates *P* < 0.05.

When we examined the cytotoxicity of the AFP-specific CTLs in *in vivo* mouse models, we found that AFP-specific CTLs expanded by DC and BA15 displayed significant tumor suppressive functions when compared with unspecific activated CTLs and control group treated with PBS. CTLs expanded by BA15 were the most efficient in these groups (Figure 5A). Hematoxylin and eosin (HE) staining showed that significantly more tumor cells exhibited morphological changes indicative of apoptosis and necrosis in the BA15 group than others. The infiltration level of CD8+ CTLs in the BA15 group was also significantly higher than that in other groups (*P* < 0.01) (Figure 5B).

## DISCUSSION

In this study, we have reported on the development of a novel AFP-specific aAPC based on the human B lymphoma BJAB cell line. We contend that this system could provide a robust platform for the generation of functional AFP-specific CTLs for adoptive immunotherapy of HCC. The aAPC we developed is a stably engineered cell line and can be generated in a standardized manner.

aAPCs have been developed as potent tools for generating and expanding antigen-specific CTLs for adoptive immunotherapy [20–23]. Although acellular synthetic aAPCs can provide good control of signal delivery and efficiently induce high T cell expansion rates, their application is hampered by several limitations [24–26]. The surface-bound antibodies of aAPCs are not only different from those provided by natural ligands but also technically difficult to stably link to the platform. Control of local release of cytokines is also difficult in acellular aAPC systems.

Cellular aAPCs derived from genetically engineered cell lines provide even easier control of signal delivery than do natural APCs and can be stored for an extended time as a readily accessible source of cells for immunotherapy. The optimal design of aAPCs for practical use in adoptive immunotherapy is still being determined. A general paradigm for the design of an optimal aAPC must include 3 stimulating signals, as does the aAPC described here. (1) Activation signal 1 is mediated by the antigenic peptide-bearing MHC that bonded with the T cell receptors specific to the corresponding epitope [27–28]. BA15 cells were genetically engineered to endogenously express AFP_158-166_ peptide and to achieve a higher specific activation rate of antigen-specific CTLs than would exogenously loaded peptide. (2) Activation signal 2 is conducted by co-stimulators, such as the B7 family proteins B7.1 and B7.2 [29–31]. BA15 cells can express levels of CD80 and CD86 as high as that of their BJAB backbone (3) Cytokines synthesized and secreted by APCs function as activation signal 3 and are essential for T cell survival and shaping of immune responses [32–34]. The BA15 cells in our study were genetically engineered to endogenously express IL-15.

The aAPC we developed also met the safety requirements of for clinical administration of immunotherapy. Gamma-ray irradiation at 20 Gy completely inhibited the proliferation of BA15 cells while keeping most of them viable for 1 round of activation. All the cells died within 12 days. Importantly, we found that the expression of activation signals was not significantly affected at this dose of radiation. In this way, irradiated BA15 cells would not cause cancer to spread and would preserve their ability to activate T cells.

IL-15, a member of the common cytokine receptor γ-chain family, plays a more important role in the activation and proliferation of CTLs than does IL-2. It can promote expansion with an early differentiation phenotype and may allow greater expansion and prolonged *in vivo* persistence. A previous study reported that IL-15 enhanced the biological activity of CD8+ T cells and induced memory CD8+ T cells with high levels of CD28 [35–38]. Similarly, we found that BA15 cells also stably expressed and secreted high levels of IL-15. Although the production of IL-15 was not adequate to completely replace exogenous IL-2 to support the proliferation of T cells, it nonetheless did enhance the biological activity of the CTLs.

Our study has some limitations. First, the expression of the AFP_158-166_ epitope peptide was only analyzed qualitatively; this aspect of our study was not quantitative. We could only indirectly conclude that the presented AFP_158-166_ peptide level in BA15 cells was higher than that of DCs loaded exogenously from the observation that the BA15-activated CTLs had a significantly higher percentage of AFP_158-166_-specific CTLs than did the DC-activated CTLs. Second, our study lacked control groups to establish the advantages of IL-15 in generating and expanding AFP-specific CTLs.

In summary, we have generated BJAB-based aAPCs expressing an endogenous AFP_158-166_ epitope peptide-HLA-A*02:01 complex, CD80, CD86, and IL-15 that successfully expanded functional CTLs specific to the AFP_158-166_-restricted epitope. The CTLs generated by the genetically engineered BA15 cell line established a higher proportion of AFP-specific CTL populations and exhibited higher cytolytic activity against AFP-positive HCC cells than did CTLs obtained from peptide-pulsed DCs.

## MATERIALS AND METHODS

### Cell culture

Blood samples were obtained from healthy donors under the supervision of the Institutional Review Board of Tianjin Medical University. Donors provided written informed consent, in accordance with the Declaration of Helsinki. We selected cells with the HLA-A*02:01 phenotype using flow cytometry (FCM) and sequence-specific primed PCR. Peripheral blood mononuclear cells (PBMCs) were isolated from HLA-A*02:01 samples. The monocytes were then purified using CD14 microbeads (Miltenyi Biotec, Bergisch Gladbach, Germany) and cultured in RPMI 1640 medium supplemented with 10% FBS (Invitrogen, Frederick, MD), IL-4, GM-CSF, and TNF-α (PeproTech, Rocky Hill, NJ) to generate mature DCs.

The human HCC cell line HepG2 (AFP+, HLA-A*02:01), colorectal adenocarcinoma cell line SW480 (AFP−, HLA-A*02:01), breast adenocarcinoma cell line MCF7 (AFP−, HLA-A*02:01), lymphoblastoid cell line T2 (AFP−, HLA-A*02:01), B-cell lymphoma cell line BJAB (AFP−, HLA-A*02:01), and embryonic kidney cell line 293T were purchased from ATCC (Rockville, MD). HepG2, SW480, MCF7, and 293T cells were cultured in Dulbecco's modified Eagle medium (DMEM), while T2 and BJAB cells were cultured in RPMI 1640 (Invitrogen) supplemented with 10% FBS.

### Plasmid and lentivirus vector construction

Plasmid JA15, derived from an IL-15 expression plasmid that had been constructed and kept by our lab, was modified to code and synergistically express AFP_158-166_ epitope peptide and IL-15. The coding sequence was cloned into a lentiviral expression plasmid. 293T cells were then transfected with the lentiviral expression plasmid and Lenti-Pac HIV packaging plasmids (GeneCopoeia, Rockville, MD) to produce lentiviral particles.

### Establishment of aAPC

A stable AFP_158-166_-specific aAPC line, named BA15, was established by infecting BJAB cells with the lentiviral particles, followed by puromycin selection for 3 weeks.

#### Phenotypic analysis

We employed FCM to detect the phenotypes of APCs on DC, BA15, and BJAB cells. We used 3 fluorochrome-conjugated antibodies: anti-human CD80, CD86, and HLA-A2 (eBioscience, San Diego, CA).

#### qRT-PCR

To determine the IL-15 expression of DC, BA15 and BJAB cells by measuring mRNA levels, we used a mirVana Isolation Kit (Ambion, Grand Island, NY) to extract the total RNA from the APCs. Reverse transcription was performed with SuperScript II Reverse Transcriptase (Invitrogen). A TaqMan Gene Expression Assay (Applied Biosciences, Grand Island, NY) was used to detect and quantify IL-15. Relative expression was normalized to GAPDH as an endogenous control using the 2^−ΔΔCt^ method.

#### ELISA

To determine IL-15 secretion levels, we incubated APCs in complete medium for 24 hours. Supernatants were collected, centrifuged to remove cellular debris, and stored at −20°C. An ELISA assay for IL-15 expression was performed according to the manufacturer's protocol (R&D Systems, Minneapolis, MN).

#### Extraction of cell surface peptides

To biochemically confirm the presence of the AFP_158-166_ peptide, we washed BA15 and BJAB cells (5 × 10^9^) twice with cold PBS and exposed them to a citrate buffer (0.13 mol/L citric acid, 0.06 mol/L Na_2_HPO_4_, pH 3.0) for 5 minutes. The eluted extracts were spun and the peptide-containing supernatant was filtered with a 0.22-μm membrane filter and frozen at −20°C for storage pending further analysis.

#### Liquid chromatograph mass spectrometry of AFP_158-166_ peptide

To determine the presence of the AFP_158-166_ peptide, we analyzed the filtered peptide extract samples from the previous step using a Single Quadrupole Liquid Chromatograph Mass Spectrometer (LCMS-2020, Shimadzu, Kyoto, Japan). The peptide solutions (20 μL) were injected on a C_18_ column (5 μm, 4.6 × 150.0 mm) (Shimadzu), with a flow rate of 0.2 mL/minute at 50°C. Mobile phase A was 0.1% trifluoroacetic acid in water, and phase B was 0.1% trifluoroacetic acid in acetonitrile, with a gradient starting at 90% A to 5% A in 6 minutes and back to 90% in 8 minutes. The peptide fraction corresponding to the synthetic AFP_158-166_ peptide was collected and analyzed with mass spectrometry. Spectra were obtained using the entire 0.2-mL/minute column effluent over a mass-to-charge ratio range of 100:1 to 2000:1.

### Irradiation of aAPCs

To ensure the safety of future clinical applications in adoptive immunotherapy, we irradiated the BA15 cells with γ-rays before use. The optimal dose of irradiation was determined using 2 criteria. First, early apoptosis had to be induced in the aAPCs had to be induced so proliferation would be inhibited but the cells kept alive for a short period. Second, irradiation had to have minimal effects on the expression of HLA, co-stimulatory molecule ligands, AFP_158-166_ epitope peptide, and IL-15. BA15 cells were irradiated with ^60^Co γ-rays at 0, 10, 20, and 30 Gy before further assays. The expression of AFP_158-166_-HLA-A*02:01 complex, CD80, CD86, and IL-15 were analyzed as described in previous steps.

#### Cell viability and proliferation assay

To assay the proliferation of BA15 cells after irradiation, CFSE-stained cells were cultured in complete medium and analyzed by FCM every day for 7 days. The BA15 cells were also counted and analyzed for viability by an MTT assay every day after irradiation during the 7-day culture period.

#### Apoptosis assay

The apoptosis rates of the irradiated BA15 cells were analyzed every 3 days using the Annexin V Apoptosis Detection Kit (eBioscience) according to the manufacturer's protocol.

### Generation of AFP_158-166_-specific CTL clone

To generate the AFP-specific CTL clone, we enriched CTLs from PBMCs using CD8 microbeads (Miltenyi Biotec). The mature DCs were incubated with the HLA-A*02:01-restricted AFP_158-166_ peptide (40 mg/mL) (SBS Genetech, Beijing, China) for 4 hours in FBS-free RPMI 1640 medium. BA15 cell and peptide-pulsed autologous DCs were used as APCs to induce AFP-specific CTLs at different APC/lymphocyte ratios in the presence of 10 U/mL IL-2 (PeproTech). The CTLs were restimulated with the APCs at days 7 and 14. The CTLs were also activated with HER2/neu_369-377_ (KIFGSLAFL) peptide-pulsed DCs and BJAB cells as the control group. The activated CTLs were then stained with HLA-A*02:01-restricted AFP_158-166_ MHC Pentamer (Proimmune, Oxford, UK) and anti-CD8 antibody. The CD8 and Pentamer double-positive populations were AFP-specific CTLs.

### Proliferation of AFP_158-166_-specific CTL clone

After 3 rounds of stimulation, CFSE-stained activated CTLs were cultured in complete medium for 7 days, and their proliferation was analyzed using FCM. The number of CTLs was counted after each round of activation with APCs.

### Human IFN-γ enzyme-linked immunospot assay

To compare IFN-γ production among the AFP-specific CTLs induced with the AFP peptide-pulsed DCs, BA15, and BJAB cells, we performed an enzyme-linked immunospot (ELISPOT) assay (R&D Systems). The control cells were either untreated or treated with phytohemagglutinin. ELISPOT data were expressed as the total IFN-γ spots/10^6^ PBMCs.

### Lactate dehydrogenase cytotoxicity assay

To evaluate the specific cytotoxic effects of *in vitro*-generated AFP-specific CTLs on AFP+ HCC cells, we used a lactate dehydrogenase cytotoxicity assay. HepG2, SW480, MCF7, T2, T2 pulsed with AFP_158-166_, and T2 pulsed with HER2/neu_369-377_ were used as the target cells. CTLs activated by the different APCs were used as effector cells and were added to the target cells at different effector-to-target ratios (10:1; 20:1; 40:1). After incubation for 6 hours, cell supernatants were collected and the amount of lactate dehydrogenase in the culture medium was assessed using the CytoTox 96 non-radioactive cytotoxicity assay (Promega, Madison, WI), according to the manufacturer's instructions.

### Adoptive cell transfer

To evaluate the specific cytotoxic effects of AFP-specific CTLs on AFP+ HCC cells *in vivo*, we used HepG2 cells to establish xenograft tumors in NOD /SCID mice. Mice were maintained at the Animal Center of Tianjin Medical University (Tianjin, China). Animal experiments were carried out according to protocols approved by the Ethics Review Committee for Animal Experimentation. HepG2 cells (5 × 10^6^ in 0.1 mL PBS) were inoculated subcutaneously into the right armpit of mice. When the tumor volumes reached 100 mm^3^, the mice were randomized into 4 groups with 8 mice in each group to receive adoptive cell transfer. Nonspecific activated CTLs, DCs, or BA15-induced AFP-specific CTLs (1 × 10^8^ in 0.2 mL PBS) were transferred into the mice *via* intravenous injection on days 0, 7, and 14. PBS was used to treat the control group. On day 21, 2 mice from each group were sacrificed, and their tumors were excised for pathological analysis. The tumors were fixed in paraformaldehyde and embedded in paraffin. To evaluate histopathologic changes, 5-μm sections of tumor specimens were stained with HE. Tumor-infiltrating lymphocytes were analyzed by immunohistochemical (IHC) staining with anti-human CD8 antibody (Santa Cruz Biotechnology, Santa Cruz, CA). The other mice were sacrificed before the mean diameter of the tumor reached 20 mm, according to institutional guidelines for the termination of animal research.

### Statistical analysis

Data are presented as means ± standard deviations. A 2-tailed Student's *t* test was performed to compare the differences between groups. *P* values < 0.05 were considered statistically significant. SPSS version 17.0 (SPSS Inc., Chicago, IL) software was used for all analyses.
